# CNS Plasticity in Injury and Disease

**DOI:** 10.1155/2016/1923950

**Published:** 2016-01-10

**Authors:** Brandon A. Miller, John C. Gensel, Michael S. Beattie

**Affiliations:** ^1^Department of Neurosurgery, Emory University School of Medicine, Atlanta, GA 30322, USA; ^2^Spinal Cord and Brain Injury Research Center, University of Kentucky, Lexington, KY 40536, USA; ^3^Brain and Spinal Injury Center, UC San Francisco, San Francisco, CA 94143, USA

Plasticity is a defining characteristic of the central nervous system (CNS). The ability of the CNS to physically change over the life of the organism, including myelination, neuronal proliferation, and synaptic changes, remains a topic of research in every subdiscipline of neuroscience from molecular to developmental neuroscience. The lay public also seeks a better understanding of neural plasticity. The recent BRAIN Initiative launched by the United States government (http://braininitiative.nih.gov/about.htm) aims to improve understanding of CNS plasticity by funding projects focused on neuronal circuitry, imaging, and neural modulation.

While the CNS has incredible plasticity compared to other organ systems, it also has unique sensitivity to injury. CNS injury and disease, from developmental injuries that occur in utero to environmental insults from toxins and trauma, can have a devastating effect on the organism. Ironically, some of the very mechanisms underlying CNS plasticity confer its sensitivity to injury. For example, neurotransmitter receptors that form the basis for learning and memory can be targets of excess excitatory neurotransmitters that induce cell death in many forms of CNS injury. CNS plasticity allows for recovery from injuries by both adaptation of the organism and cellular regeneration. However, these attempts at regeneration are often incomplete and unsuccessful. This was observed by the Nobel Prize winner Santiago Ramón y Cajal in the early 20th century and continues to be the basis of ongoing research on brain and spinal cord injury.

The articles in this special issue illustrate diverse examples of CNS plasticity, both in response to injury and as avenues for recovery. The scope of diseases discussed, motor neuron disease, stroke, viral infection, and spinal cord injury, illustrates that a better understanding of basic mechanisms of neural plasticity could lead to better treatments for numerous CNS diseases.

Spinal cord injury has received much attention as a target for regenerative therapies. In their paper in the issue, V. Buzoianu-Anguiano and colleagues show that a combination of degenerated peripheral nerve and bone marrow stromal cell transplantation improves axonal regrowth and myelination after spinal cord injury. Other papers in this issue address neural plasticity in diseases where it is less frequently examined. The paper by V. Atluri and colleagues highlights the neuronal injury and synaptic changes that can occur with CNS viral infection and R. Gulino reviews the plasticity that occurs in the mouse models of motor neuron disease used to model amyotrophic lateral sclerosis in humans.

Several human subject studies in this issue highlight the fact that CNS plasticity occurs in both injury and disease and that plasticity may be a target for relatively simple and potentially cost-effective therapies. A. Green and colleagues demonstrate changes in the motor cortex due to chronic spinal cord compression and G. Jiang and colleagues show that cortical changes occur after limb amputation. C. Pin-Barre and J. Laurin's review of exercise as a therapy for stroke discusses how endogenous plasticity can be enhanced by physical exercise. Similar findings from M. Weinstein and colleagues show that bimanual therapy can yield both functional and radiological changes in children with hemiparesis resulting from perinatal birth injury. These papers illustrate that treatments, including physical therapy, hold promise by increasing endogenous CNS plasticity. However, R. Brunkhorst and colleagues demonstrate that functional improvements are not always coincident with histological recovery thereby reinforcing the complex nature of CNS plasticity.

Collectively, this special edition highlights the therapeutic and mechanistic importance of understanding and manipulating CNS plasticity in both animal models and human disorders.

